# Phenolics as GABA_A_ Receptor Ligands: An Updated Review

**DOI:** 10.3390/molecules27061770

**Published:** 2022-03-08

**Authors:** José-Luis Ríos, Guillermo R. Schinella, Inés Moragrega

**Affiliations:** 1 Departament de Farmacologia, Facultat de Farmàcia, Universitat de València, Av. Vicent Andrés Estellés s/n, Burjassot, 46100 Valencia, Spain; 2 Facultad de Ciencias Médicas, Universidad Nacional de La Plata, La Plata BA1900, Argentina; schinell@gmail.com; 3 Instituto de Ciencias de la Salud, UNAJ-CICPBA, Florencio Varela BA1888, Argentina; 4 Departament de Psicobiologia, Facultat de Psicologia, Universitat de València, Av. Blasco Ibáñez 21, 46010 Valencia, Spain; ines.moragrega@uv.es

**Keywords:** phenolics, flavonoids, phlorotannins, GABA, CNS pathologies, GABA_A_R modulators

## Abstract

Natural products can act as potential GABA modulators, avoiding the undesirable effects of traditional pharmacology used for the inhibition of the central nervous system such as benzodiazepines (BZD). Phenolics, especially flavonoids and phlorotannins, have been considered as modulators of the BZD-site of GABA_A_ receptors (GABA_A_Rs), with sedative, anxiolytic or anticonvulsant effects. However, the wide chemical structural variability of flavonoids shows their potential action at more than one additional binding site on GABA_A_Rs, which may act either negatively, positively, by neutralizing GABA_A_Rs, or directly as allosteric agonists. Therefore, the aim of the present review is to compile and discuss an update of the role of phenolics, namely as pharmacological targets involving dysfunctions of the GABA system, analyzing both their different compounds and their mechanism as GABAergic modulators. We focus this review on articles written in English since the year 2010 until the present. Of course, although more research would be necessary to fully establish the type specificity of phenolics and their pharmacological activity, the evidence supports their potential as GABA_A_R modulators, thereby favoring their inclusion in the development of new therapeutic targets based on natural products. Specifically, the data compiled in this review allows for the directing of future research towards *ortho*-dihydroxy diterpene galdosol, the flavonoids isoliquiritigenin (chalcone), rhusflavone and agathisflavone (biflavonoids), as well as the phlorotannins, dieckol and triphlorethol A. Clinically, flavonoids are the most interesting phenolics due to their potential as anticonvulsant and anxiolytic drugs, and phlorotannins are also of interest as sedative agents.

## 1. Introduction

The γ-aminobutyric acid (GABA) was discovered in the mammalian brain in 1950 by Jorge Apawara [1], Eugene Roberts and Sam Frankel [2], although it was previously synthesized in 1883 when it was only known as a metabolic product in Krebs’ cycle of microorganisms and plants. GABA is the main inhibitory neurotransmitter in the central nervous system (CNS) although it is present to a lesser extent in the peripheral nervous system. Since this neurotransmitter has a fundamental role in the regulation of excitatory and inhibitory processes in the brain, any disruption can cause CNS pathologies [3]. GABA is currently known to be involved directly or indirectly in CNS diseases including anxiety [4], cognitive [5] and sleep disorders [6], epilepsy [7], depression and bipolar disorder [8], schizophrenia [9], Alzheimer's [10] and Parkinson’s diseases [11], memory impairments [12], eating disorders [13], response to anaesthesia in surgical operations [14], etc. Thus, a major aim of therapeutic approaches targeting the GABA system, on which it depends for neuronal inhibition, is to recover the neuronal balance between excitation and inhibition lost in all these pathologies. Importantly, GABA is also critical in neuro-developmental disorders such as autism spectrum disorders or Angelman syndrome due to its pivotal role in the performance of central functions in the development and function of the brain such as neurogenesis, neuronal maturation and apoptosis [15]. In addition, GABA influences neural migration, acts as a neurotrophic factor, and facilitates neurite extension [16]. Recently, it has revealed its relevant implication in the myelinization and maturation of oligodendrocytes and Schwann cells [17], and seems to express differently in the brain depending on the developmental stages [18].

**Figure 1:**
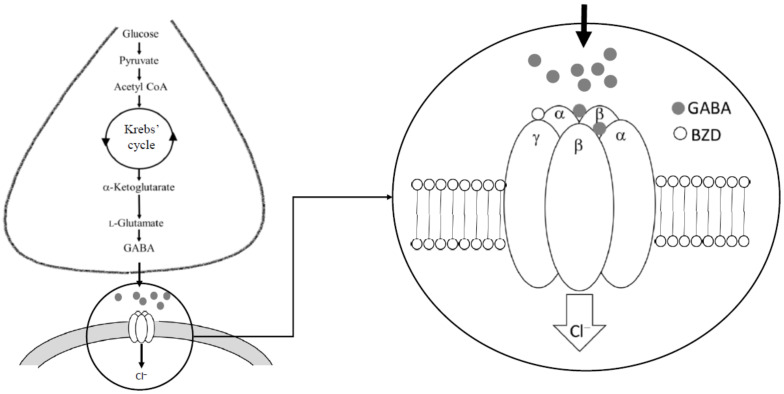
Synthesis of GABA and the GABA_A_ receptor with agonist and antagonist binding sites.

The GABAergic system is mainly composed of GABA, GABA transporters (GATs), GABA receptors (GABA-Rs), and GABAergic neurons and neuroglia, mainly astrocytes involved in its metabolic regulation [15]. GABA neurons are widely distributed in the brain and are localized in axonic terminals, somas, and to a lesser extent in dendrites. Therefore, its expression is highly correlated with GABA levels and its subsequent GABAergic neurotransmission at the inhibitory synapse [19]. In the mammalian brain, the synthesis of GABA occurs via a metabolic pathway referred to as the GABA shunt, a closed-loop system in charge of the dual task of production and conservation of GABA supply. The main precursor for GABA synthesis is glucose, although other aminoacids and pyruvate can take this role. The first step in the GABA shunt is transamination of the α-ketoglutarate produced by the metabolism of glucose in the Krebs’ cycle by the glutamate dehydrogenase, producing L-glutamate, which must be decarboxylated by glutamic acid decarboxylases (GADs) to form GABA (Figure 1). GAD includes two isoforms expressed by separate genes which have different regulatory processes and molecular properties: GAD-65, involved in synaptic transmission and plasticity, and GAD-67, which controls the metabolic GABA synthesis [15]. GAD enzymes need pyridoxine (vitamin B_6_) as a cofactor, which is only expressed in cells using GABA as a neurotransmitter; therefore, it is used as a marker to locate GABA neurons along the CNS [20,21].

The metabolization of GABA by GABA transaminase (GABA-T) is the next step in the GABA shunt. A molecule of GABA can be metabolized by the transamination reaction catalysed by GABA-T only if a molecule of precursor, such as α-ketoglutarate, is present to accept the amino group removed from GABA, thereby reforming the glutamic acid [22]. GABA is loaded into synaptic vesicles by a vesicular neurotransmitter transporter and is liberated from nerve terminals by calcium-dependent exocytosis. However, non-vesicular forms of GABA secretion (for example, by reverse transporter action) have also been described, which might be particularly important during brain development [18]. In addition, GABA can also modulate its own release in the pre-synapse through auto-receptors, resulting in release inhibition. After its synthesis, release, and binding to receptors, the GABA signal is terminated and recaptured by the glia and by presynaptic nerve terminals to be synthesized again and/or into surrounding glial cells by a class of plasma-membrane GATs that regulate the duration of action and release of GABA into extracellular space in a Ca^2+^-dependent manner [23].

GABA-Rs can be separated into two major types: metabotropic (GABA_B_Rs) and ionotropic (GABA_A_Rs) families that function differently. GABA_A_Rs are ligand-gated ion channels permeable to Cl^−^ and HCO_3_^−^ anions which hyperpolarize the neuron, thereby obtaining a rapid inhibitory effect. They commonly include GABA_A_Rs and GABA_C_Rs, based on their ability to form heteromeric and homomeric receptors, respectively; however, more recently, GABA_C_Rs have been classified as a subtype of GABA_A_Rs [18]. GABA_A_Rs are composed of five subunits arranged around of a central pore that constitutes the actual ion channel that mediates the major form of fast inhibitory neurotransmission in the CNS [24]. There are at least 19 distinct GABA_A_R subunit genes [25,26]. This diversity results in different homomeric or heteromeric subunit combinations so that even if the combinations are restricted only to the more common ones (those containing two α, two β and another subunit γ), more than 2000 different GABA_A_Rs could exist [3]. 

It is known that most neurons express GABA_A_Rs, which are the most important for pharmacological modulation [26]. However, the subunit profile of GABA_A_Rs is important for physiological action, and depends on several factors including brain region, cell type, developmental stage, and physiological or pathophysiological conditions [27]. They consequently have a different pharmacology and distinctive functional characteristics [28], modulating different functions in the brain. Remarkably, the location of these receptors is also a determinant of their inhibitory activity. For instance, whereas GABA_A_Rs composed of α_1_βγ_2_, α_2_βγ_2_, α_3_βγ_2_ are primarily located synaptically and contribute to phasic inhibition, those receptors composed of α_4_βγ_2_, α_5_βγ_2_, α_6_βγ_2_, or αβε subunits are partially placed extrasynaptically, hence contributing to both phasic and tonic inhibition [29]. On the contrary, GABA_A_Rs composed of αβ or αβδ subunits seem to be exclusively located extrasynaptically and hence only exert tonic inhibition on neurons [25].

Although several binding sites at the GABA_A_Rs have been recognized, the total number of allosteric binding sites remains to be established [30]. Currently, a variety of compounds selectively modulating GABA_A_Rs have been identified, although incompletely, since they can also modulate other subtypes in similar concentrations [25]. The GABA_A_R binding sites most studied in vitro utilizing the radioligand binding technique are the GABA/muscimol, the benzodiazepine (BZD), and the *tert*-butyl bicyclephosphorothionate/picrotoxin binding sites. GABA_A_Rs can also be blocked selectively by the alkaloid bicuculline [31]. However, GABA-Rs incorporate many allosteric modulatory sites, agents known to modulate GABA_A_R, whose number is constantly increasing, although the most tested ligand bindings are BZDs, alcohol, barbiturates, neurosteroids, anaesthetics, and anticonvulsants, besides some natural products such as flavonoids, amongst others [26,32]. As already mentioned, GABA_A_Rs with different subunit compositions exhibit different pharmacological and channel-gating properties. Furthermore, they are expressed differently during development than in the adult brain [18], and finally, they are also accumulating on different neuronal cell surfaces and are subject to differential regulation by extracellular cues [26]. Therefore, the effects of BZDs on GABA-Rs are complex and dependent on the receptor subunit composition. The pharmacology of BZD-receptor subtypes is mainly determined by α and γ isoforms forming this site, whereas the channel-building β subunits do not greatly affect the sensitivity of GABA_A_Rs to BZD ligands [31]. Recently, the use of transgenic mice has expanded our knowledge of the physiological role of the different α subunits, and enabled the identification of diazepam's effects that are missing or reduced in mutant mice [31]. These models have demonstrated that α_1_βγ_2_ receptors mediate sedation, anterograde amnesia and some anticonvulsant properties of diazepam. However, α_2_βγ_2_ principally mediated the anxiolytic effects and muscle relaxation of diazepam; therefore, these actions can be separated in molecular terms due to their mediation by different pathways [26]. In turn, α_3_ seems also to be implicated in sedation [31] and anxiolytic effect [33] given its recently discovered role in epileptic seizures, dysmorphic features, intellectual disability, and developmental delays [34,35]. For their part, α_5_βγ_2_ receptors are involved in learning and memory processes [36,37].

Some natural products such as terpenoids, polyacetylenic alcohols and certainly flavonoids have demonstrated effects on the GABA system [30], for example, some members of the flavonoid family have shown moderate binding affinities for the BZD-site, thereby positioning themselves as potential targets that could avoid unwanted effects provoked by BZDs. Therefore, the aim of the present review is to highlight and discuss the role of phenolics as GABA modulators, analyzing the different compounds and their mechanism as GABAergic agents. We focus this review on articles written in English since the year 2010 cited in the PubMed and Web of Science databases as well as the Cochrane Central Register of Controlled Trials. The key words used were ‘phenolics’, ‘flavonoids’, ‘polyphenols’, and ‘GABA’. In the last review of this subject (31 December 2021), we found 677 articles in the last ten years, of which 174 had been published in the last five years; of these, 75 articles had been published in the last twelve months. Regarding experimental studies, the authors used in vitro or in vivo experiments, and some included in silico protocols. Despite the large number of publications, only 23 of these 677 articles are clinical trials.

## 2. Natural Products as Potential GABA Modulators

The interest in natural products as modulators of the GABAergic system is a response to the need to finding new active compounds different to BZD, thereby avoiding its wide range of undesirable effects, such as tolerance, abstinence, dependence, and even memory impairment. For this reason, many researchers have focused on this subject, although most studies targeted different extracts of plants with known activity on the CNS. Some authors tested different types of extracts [38,39,40] and essential oils [41,42] as potential sources of active agents in animals [42,43], and humans [40], and other authors studied their possible pharmacological potential in silico [42,44,45]. Of all the tested compounds, phenolics were of greatest interest [46], especially the flavonoids [26,31,47] and some types of tannins [48,49,50,51].

### 2.1. Phenolics as Potential GABAergic Compounds

Phenolics, which very common in the plant kingdom, are found in variable amounts depending on the taxonomic group (family, genus, and species). This broad group consists of different structural types, highlighting flavonoids, tannins, phenolic acids, stilbenes and lignans, among others [52,53]. Many scientific publications conclude that the in vitro antioxidant activity of flavonoids is a relevant pharmacological property demonstrated by this phytochemical group [54]. However, a direct relationship between in vitro antioxidant capacity and pharmacological activity is questioned by different authors [55,56,57]. At present, there is convincing evidence that the mechanisms by which flavonoids exert their pharmacological effects are not simply due to their redox properties, but rather to their ability to bind directly to target proteins or peptides that regulate different cellular functions. Active flavonoids and other phenolics found in medicinal plants modulate the activity of a large class of enzymes and receptors [53,58].

### 2.2. Flavonoids and GABA_A_ Receptors

In the case of flavonoids, despite their well-established role as antioxidants, recent evidence shows their direct interaction with proteins, making them ideal small molecules for the modulation of enzymes, transcription factors, and receptors, and thus they can act in different ways in several diseases [58]. For example, flavonoids can be antimicrobial [59,60,61], hepatoprotective [62], coronary heart disease preventive [62], anti-inflammatory [63,64], neuroprotective, [65] and can also have antiviral [66], antitumor, and anticancer properties [67,68]. Table 1, Table 2, Table 3, Table 4 and Table 5 summarize the principal studies of flavonoids, including chemical structure and relevant data obtained by the studies compiled in the present review.

As previously mentioned, many natural and synthetic flavonoids have been identified as influencing ionotropic GABA-R. The papers reviewed include different areas of interest with regard to the function of the potential mechanisms of flavonoids. For example, some of them show interesting subtype selectivity on GABA_A_R complexes in vitro, results consistent with their in vivo effects. They can act as second-order modulators of first-order modulation by BZDs and modify the flumazenil-insensitive modulation of the GABA_A_R function [31]. Among the relevant flavonoids with activity on GABA-Rs, different publications highlight the role of flavones (apigenin, chrysin), biflavones (amentoflavone) and flavonols (baicalin) [25,47,83,99,121], epicatechin and its derivatives [122], chalcones [117], etc. The number of active compounds and the knowledge of the relationship between chemical structure and GABAergic activity increased through the use of quantitative structure–activity relationship (QSAR) models. This in silico protocol calculates the binding affinity constants of a great number of natural and synthetic flavonoids [123]. These structure-activity relationship studies showed that the incorporation of electronegative groups into the C-6 and C-3′ on the flavone backbone increases affinity with the BZD binding site [32]. A 2′-hydroxyl on flavonoid was also found to be relevant for its BZD binding affinity [26,32,123]. In the case of in vivo experiments with different animal models, flavonoids modify sleep directly or indirectly, acting in different processes related to the CNS. Flavonoids were also demonstrated to be a safe natural alternative treatment for neuropathic pain, oxidative stress, and neuroinflammatory diseases [124]. 

Therefore, flavonoids are considered multi-target drugs exhibiting a wide range of actions. Consequently, this review highlights flavonoids described in the last 10 years as GABA_A_ modulators and their effects on CNS. Some flavonols showing affinity to the GABA_A_-BZD site have also been studied [47,125]; of these, quercetin (Table 1), is probably the most widely investigated. It is a natural flavonoid extensively distributed in the plant kingdom with redox properties [126] and antagonistic effects to GABA_A_ρ_1_Rs [127]. Like many other ionic channels, GABA_A_ρ_1_Rs can be modulated by several reducing and oxidizing agents [128]. However, whether quercetin's effects on GABA_A_ρ_1_Rs are mediated by a redox mechanism or by an allosteric interaction (or both) was not established. Quercetin as a negative allosteric modulator of GABA_A_Rs exerted antipsychotic activity, facilitating further therapeutic development in excitatory-inhibitory imbalance disorders [69]. Taken together, recent results suggest that quercetin's antagonistic actions on GABA_A_ρ_1_Rs are mediated through a redox-independent allosteric mechanism [70]. Also, quercetin affects the expression of the GABA_A_α_5_R, which could be a mechanism for reducing seizure severity (at anticonvulsive doses) or even could be a marker of seizure severity [71]. However, the anxiolytic-like effect of rutin (quercetin-3-*O*-rutinoside, Table 1) involves GABAergic neurotransmission not associated with BZD-Rs [76].

A similar compound, myricetin (5-hydroxy quercetin, Table 1), enhances GABA_A_R activity via the Ca^2+^ channel/calmodulin-stimulated protein kinase II dependent mechanism, which is distinctively different from that of most known BZD-binding site agonists of GABA_A_R [74]. Recently, Sun et al., (2019) demonstrated that myricetin regulates the brain-derived neurotrophic factor/tropomycin receptor kinase B and the expression of matrix metallopeptidase-9. Myricetin also restores the expression of GABA_A_R and GAD-65, as well as the glutamate/GABA balance [129]. Its dihydroderivative (dihydromyricetin) competitively inhibited BZD-site [^3^H]-flunitrazepam binding, suggesting that the known interaction of dihydromyricetin with ethanol involves the BZD sites on GABA_A_Rs [109]. Furthermore, dihydromyricetin had no adverse side effects on pregnant rats, which could make it a good candidate for prevention of foetal alcohol spectrum disorders [110]. More recently, dihydromyricetin was demonstrated to potentiate GABAergic activity, as shown in electrophysiology studies of α_5_β_3_γ_2_ GABA_A_Rs expressed in *Xenopus* oocytes, although its metabolite 4-*O*-methyl-dihydromyricetin negatively modulates GABAergic activity [130]. In the case of the quercetin 5-dehydroxy-derivate fisetine (Table 1), chronic treatment can delay or correct neuropathic hyperalgesia and allodynia in mice with type-1 diabetes; mechanistically, this effect may be associated with its antioxidant activity and spinal GABA_A_ [73]. In the case of the 3′-dehydroxy-3,6-dimethoxy derivative of quercetin, viscosine's (Table 1) anxiolytic and anticonvulsant effects are mediated via its positive allosteric modulatory action of GABA at different GABA_A_R subtypes [75].

Flavones are an extensive and relevant group of flavonoids, many of which also have an effect on GABA_A_Rs, which can be regulated by this kind of flavonoid. One the one hand, for example, baicalin, wogonin and baicalein (Table 2), from *Scutellaria baicalensis* Georgi (Lamiaceae), have been reported to bind to the GABA_A_-BZD receptor. Wogonin and baicalein binds the BZD site on the GABA_A_Rs stronger than baicalin [90]. On the other hand, the anticonvulsant effects of baicalein were inhibited by flumazenil, therefore the anticonvulsant effect is mediated by the BZD binding site of GABA_A_R, and it is related to the 5,7-dihydroxyl structure. Otherwise, other similar compounds present in this plant were also tested: baicalin and oroxylin A (Table 2) also showed anticonvulsant activity whereas the 6-methoxy had no effect. Yoon et al., (2011) concluded that the 5,7-dihydroxy group might be important for convulsion-related activities but that the 6-methoxy group is not relevant as an anticonvulsant [87]. In the case of chrysin (6-dehydroxy baicalein, Table 2), this flavone produced anxiolytic-like effects via GABA_A_Rs in a model of surgical menopause in rats [84], and prevented anxiety-like behaviour in two unconditioned models used to evaluate anxiety-like behaviour; these effects were mediated by actions on GABA_A_Rs [83]. Moreover, luteolin (3′,4′-dihydroxy derivative of chrysin, Table 2) ameliorates mechanical and cold hyperalgesia at least in part by activating GABA_A_Rs in a flumazenil-insensitive manner [79]; it also has negative modulatory effects on both recombinant and endogenous GABA_A_Rs and inhibits phasic rather than tonic inhibition in the hippocampus [80]. The 4′-hydroxy derivative of luteolin, the apigenin (Table 2) increases pentobarbital-induced sleep behaviours through chloride ion channel activation [77], whereas its glucosides (vitexin, isovitexin, and spinosin, Table 2) modulate GABA_A_R via the BZD-binding site, thus exerting their memory-enhancing and anxiolytic-like effects [104].

In the case of polymethoxyflavones (Table 2), both the 5- and 6-methoxy derivatives are described as positive allosteric modulators of GABA_A_Rs [93,131]. Also, different polymethoxyflavones exhibit anxiolytic activity without altering locomotor responses; the participation of GABA_A_Rs in the action of some of these compounds was demonstrated by Shajib et al., (2018). Specifically, compound 3,5,6,7,8,3′-hexamethoxy-4′,5′-methylenedioxyflavone, 6,7,4′,5′-dimethylenedioxy-3,5,3′-trimethoxyflavone, and 3,5,8,3′,4′,5′-hexamethoxy-6,7-methylenedioxyflavone showed anxiolytic-like activity, with the involvement of GABA_A_R in the latter two compounds, as was demonstrated in the reversal effects of flumazenil, whereas the first and third showed anxiolytic activity without modifying locomotor responses [98]. Liu et al., (2018) isolated 28 flavonoids from two Tibetan medicinal plants, and the analysis by structure-activity relationships indicated that 6- and/or 8-methoxy flavones have the highest binding affinity to GABA_A_Rs. Furthermore, the compound 5,7,2′,4′-tetrahydroxy-6,5′-dimethoxyflavone, which has an IC_50_ value of 0.10 μM in binding affinities to GABA_A_R, presented high anticonvulsant activity against chemical-induced and electrogenic seizures, without myorelaxation or sedation [99].

The dihydro-derivatives of flavones constitute a special group of flavonoids called flavanones (Table 3). The hydrogenation of ring C changes planarity to a special conformation. In the case of the dihydro-derivative of apigenin, the compound called naringenin has two possible configurations: *R* and *S*, given the corresponding (+)- or (−)-naringenin, the (2*S*)-5,7,4′-trihydroxyflavan-4-one being the more frequent natural product, although it racemizes quickly. Copmans et al., (2018) studied the effects of naringenin (Table 3) and its methylated derivatives naringenin 7-*O*-methyl ether and naringenin 7,4′-dimethyl ether in zebrafish and mouse models, observing that the methylated derivatives are highly effective against pentylenetetrazole-induced seizures in larval zebrafish, whereas naringenin had only limited activity. The molecular mechanisms by which these compounds exert their activity are substantially different from the parent compound naringenin, with only mild affinity for the GABA_A_-BZD receptor site, perhaps indicating that its activity is not only due to this mechanism but other anticonvulsant mechanisms such as the antagonism of the glutamatergic system which could predominate over the reduction of GABA_A_Rs [132]. Some glycosidic forms of flavanones have also been described as potential GABAergic agents. For example, the anticonvulsant activity of hesperidin, hesperetin 7-*O*-rutinoside (Table 3), could mediated by the modulation of GABA-BZD receptor [111], and the antinociceptive effects demonstrated in postoperative pain conditions by eriocitrin (eriodictyol 7-*O*-rutinoside, Table 3) could be mediated through opioid and GABA_A_Rs [114].

Different prenylflavonoids are highly interesting as GABA modulators. For example, a study with radioligand binding and docking indicates a possible dual mode of action in the case of 6-prenylnaringenin (Table 3) on GABA_A_Rs. Indeed, it may be a positive allosteric modulator at the α+ β- binding interface as well as a null modulator at the flumazenil-sensitive α+ γ_2_- binding interface [107]. In the case of xanthohumol (prenylchalcone, Table 5), isoxanthohumol (prenylflavanone) and 8-prenylnaringenin, these flavonoids potentiated GABA-induced displacement of [^3^H]-ethynyl bicycloorthobenzoate radio ligand binding in a concentration-dependent manner, with an IC_50_ for GABA_A_Rs of 29.7, 11.6, and 7.3 µM, respectively [108]. Another interesting group of prenylated flavonoids are the lavandulyl derivatives. The main ones studied are kushenol I, sophoraflavanone G, (−)-kurarinone, and kuraridine, which have been shown to be GABA_A_R modulators [106]. Another chalcone, isoliquiritigenin (Table 4), also studied for its affinity for GABA_A_-BZD, showed a 65 times higher affinity for these receptors than diazepam, with a dissociation constant of 0.4 nM. The effect on GABA currents was blocked by flumazenil and ZK-93426 (a weak partial inverse agonist of BZD receptors); results indicate that the hypnotic effects of isoliquiritigenin occur by a positive allosteric modulation of GABA_A_-BZD receptors [117]. Ferreira et al., (2021) synthesized four halogenated chalcones and studied the relation between these compounds and GABAergic neurotransmission in the modulation of anxiolytic and anticonvulsant activities; they then established the possible direct interactions of these molecules with GABA_A_Rs through a molecular docking study. Pre-treatment with the synthetic chalcones reduced the convulsions induced by pentylenetetrazole and were completely antagonized by flumazenil, which implies the involvement of GABA-Rs, which was confirmed by binding/activity assays. Finally, the authors demonstrated the structure–activity relationship, in which the pattern of substitution is highly relevant to the intensity of convulsive activities, the 2,4-dichloro derivatives being the principal active compounds, whereas the 1-fluorine substituted has higher efficacy than the 4-fluorine derivative. Importantly, none of them had toxic effects on CNS [133].

Another group of interest is the dimeric forms known as biflavonoids. Three compounds: mesuaferrone B, rhusflavone, and agathisflavone (Table 5) competitively inhibited flumazenil binding with a K_i_ of 0.280, 0.045, and 0.091 μM, respectively. Of these, rhusflavone is the most relevant one, since it induces sleep via positive allosteric modulation of GABA_A_-BZD receptors. The presence of conjugated ketone and C6-C8” linkage in this biflavonoid may be responsible for decreasing sleep latency and increasing sleep duration through the BZD-site of the GABA_A_R [120].

The principal flavonoid in green tea is (−)-epigallocatechin-3-*O*-gallate (Table 4) which enhances hypnotic effects in pentobarbital-treated mice by increasing the sensitivity of GABA-Rs to pentobarbital and decreasing the α-subunit expression without effects on the expression of β- and γ-subunits. A second mechanism could be the induction of Cl^−^ influx by hyperpolarization of the membrane, which might be relevant in pentobarbital-induced sleeping behaviours [119].

### 2.3. Phlorotannins as Modulators of GABA_A_Rs

**Figure 2:**
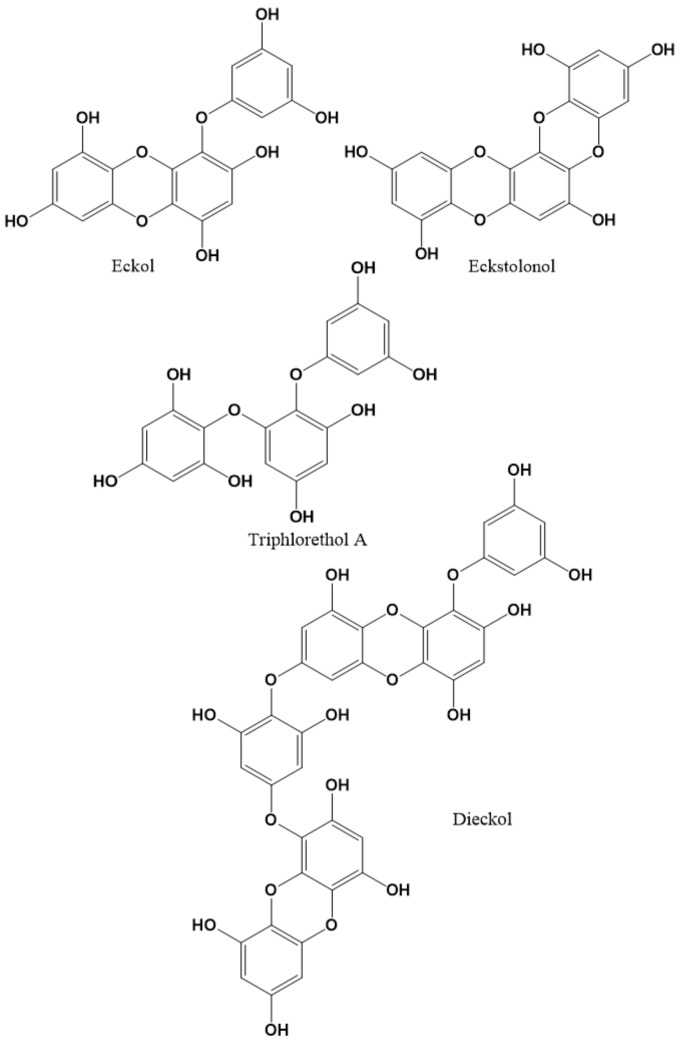
Chemical structures of phlorotannins studied as modulators of the GABA_A_ receptor.

The first reference to phorotannins dates back to 1970, when they were known as Phaeophyta tannins, marine algal polyphenols or polyphloroglucinols, but were subsequently unified as phlorotannins due to their characteristics and reactivity similar to terrestrial tannins [134,135,136]. Their chemical structures are derived from phloroglucinol (1,3,5-trihydroxy benzene); condensed in different units, it yields oligomers from three to eleven units, with bonds between both C-C and C-O atoms. From this type of molecular union derive different types of subgroups. An example are phloroethols, in which phloroglucinol residues are interconnected in the *ortho*-, *meta*- or *para*-position as ether bonds. Fuhalols and eckols have an additional hydroxyl group on the terminal monomer unit, and this last group (eckols) contains a 1,4-dibenzodioxin group in its structure. The carmalol group contains a unique set of phlorethols with different substitution patterns, and the fucophloroethols are formed by the combination of ether and phenyl. Other chemical structures (fucophloroeckols and fucofuroeckols) are heterocyclic bonds [134,136]. In several investigations, phlorotannins have demonstrated different biological and pharmacological properties, including neuroprotection [137]. For example, to evaluate the potential of this type of compound, Cho et al., (2012) tested 30 ethanol extracts from seaweed (eight green seaweeds, 11 red seaweeds, and 11 brown seaweeds from the Japanese and Korean coasts) in a GABA_A_–BZD receptor binding assay and a pentobarbital-induced sleep test for their potential sedative–hypnotic properties through their binding activity to the GABA_A_–BZD receptor. Of these, *Ecklonia cava* Kjellman (Lessoniaceae) was selected for having the highest binding activity (IC_50_ 0.1269 mg/mL), and its ethanol extract was investigated in vivo (1000 mg/kg). The results showed that it prolonged sleep duration induced by pentobarbital (45 mg/kg, i.p.), up to 142 min, in a similar level to diazepam, whereas with a sub-hypnotic dose of pentobarbital (30 mg/kg, i.p.), the extract increased the rate of sleep onset dose-dependently (92%). The ethanol extract was liquid-liquid fractioned in four solvent fractions and the ethyl acetate fraction had the highest sedative properties. This extract principally contains phlorotannins, which were tested for [^3^H]-flumazenil binding: eckol, eckstolonol, dieckol, and triphlorethol-A (Figure 2) showed the best binding affinity (K_i_ values were 1.070, 1.491, 3.072, and 4.419 μM, respectively). The hypnotic effects of the ethanol extract and its ethyl acetate fraction were fully inhibited by a specific GABA_A_–BZD receptor antagonist (flumazenil), which indicates that phlorotannins of *Ecklonia cava* induce sleep by positive allosteric modulation of the GABA_A_–BZD receptor [48].

The same authors (Cho et al.) studied the depressive effects of a polyphenol-rich enzymatic extract (obtained after digestion with Celluclast from Novo Nordisk) using a picrotoxin-induced seizure test and a pentobarbital-induced sleep test, obtaining significant anticonvulsive and sleep-inducing effects, but only at doses higher than 500 mg/kg. In the assay of the phlorotannin-rich fraction obtained from the polyphenol-rich enzymatic extract, it was observed that the pentobarbital-induced sleep was potentiated at doses above 50 mg/kg. The same extract showed binding activity on GABA_A_-BZD receptors, and the sleep-inducing effects of both extracts and the positive control diazepam were completely blocked by flumazenil. These results confirm the depressive effects of phorotannins on CNS by a positive allosteric modulation of GABA_A_-BZD receptors such as diazepam (GABA_A_-BZD agonist) [49].

In a third study, Cho et al., (2014) tested the effects of a phlorotannin extract and its major constituent eckstolonol (Figure 2) after oral administration on sleep-wake profiles in mice (C57BL/6N) using diazepam as a positive control. To study these effects and the hypnotic mechanism, they used electroencephalograms (EEG) and electromyograms, and flumazenil, as a GABA_A_-BZD receptor antagonist. Both the phlorotannin extract (>250 mg/kg, p.o.) and eckstolonol (>12.5 and 50 mg/kg, p.o.) decreased sleep latency and increased the amount of non-rapid eye movement (NREM) sleep. Both had an effect on EEG power density of NREM sleep, and their hypnotic effects were completely abolished after treatment with flumazenil [50]. In another contribution, Kwon’s team studied a standardized phlorotannin supplement on sleep-promoting effects via GABA_A_-BZD in mice with effects similar to those described previously. They also investigated the effect on caffeine-induced sleep disruption in mice (25 mg/kg, p.o.) by analysing sleep architecture based on EEG and electromyogram findings, using zolpidem as a positive control. The phlorotannin extract (500 mg/kg) attenuated caffeine-induced sleep disruption and inhibited the arousal effects of caffeine without any changes in delta activity during NREM sleep, whereas zolpidem (10 mg/kg) also attenuated the process but produced a decrease in delta activity [51]. A complementary study by Kwon et al., (2019) identified the compound dieckol (Figure 2) as being responsible for these effects due to its properties as an allosteric activator of the GABA_A_-BZD receptor. Specifically, using the whole cell patch clamp technique, phlorotannins enhanced the activity of GABA_A_-BZD receptors in a heterologous system and in primary cultured neurons. In the case of the isolated principle dieckol, it increased the GABA_A_R-mediated inward current in HEK293T cells containing the α_1_ subunit in a dose-dependent manner, and increased the amplitude of GABA_A_-BZD receptors in primary cultured neurons. These effects were blocked by co-treatment with flumazenil, thereby corroborating that phlorotannins in general and dieckol in particular act as positive allosteric activators of GABA_A_-BZD receptors, which justifies the mechanism as sedative-hypnotic [138]. This activity was ratified in vivo by Yoon et al., (2020) which evaluated the effect of dieckol on the sleep-wake state of mice by analysing EEGs and electromyograms, which revealed that it accelerated initiation and increased NREM sleep duration and shortened sleep latency. Dieckol was also evaluated by EEG power density, and did not affect sleep intensity, while zolpidem reduced it. Finally, the sleep-enhancing effect of dieckol and zolpidem in mice treated with zolpidem or dieckol plus flumazenil was inhibited, which confirms that dieckol exerts sleep-enhancing effects by activating the GABA_A_-BZD receptor [139].

Triphlorethol A (Figure 2) is another phlorotannin of interest investigated by Yoon et al., (2018). Its effect on the sleep-wake architecture and profiles was evaluated based on EEG and electromyogram data from mice (C57BL/6N) using zolpidem (10 mg/kg) as a positive control. Triphlorethol A (5, 10, 25, and 50 mg/kg, p.o.) decreased sleep latency in a dose-dependent manner and increased the sleep duration induced by pentobarbital. At the higher dose, triphlorethol A decreased sleep latency and increased the amount of NREM sleep in mice without affecting rapid eye movement (REM) sleep. Triphlorethol A had no effect on the delta activity of NREM sleep, whereas zolpidem decreased it [140]. 

Taken together, these results support the sleep-promoting effects of phlorotannins, namely in three compounds of interest such as eckstolonol, dieckol and triphlorethol A, to develop novel sedative hypnotic drugs. However, the number of studies in humans is quite limited, with only one relevant trial reported by Um et al., (2018). They carried out a randomized, double-blind, placebo-controlled trial with 24 patients that consumed either a placebo or phlorotannin supplement (500 mg/day, for 1 week, 30–60 min prior to bedtime). Characteristic and sleep parameters were assessed at baseline and after one week with sleep questionnaires and polysomnography. At the end of the treatment, 20 patients had completed the sets of sleep parameters. The results showed that phlorotannins increase sleep duration scores vs. placebo with no significant differences in total Pittsburgh Sleep Quality Index scores. Wakefulness after sleep was lower in the phlorotannin group vs. placebo as well as total wake time as was observed by polysomnography. Moreover, the respiratory disturbance index during supine REM sleep was significantly lower in the phlorotannin group, which also had no serious adverse effects. This clinical trial supports the idea of the potential of these compounds for future studies, since there is sufficient evidence and the mechanism is clear. Only pharmacokinetic studies and their potential negative effects remain to be considered [141].

### 2.4. Other Types of Compounds Active on GABA_A_Rs

**Figure 3:**
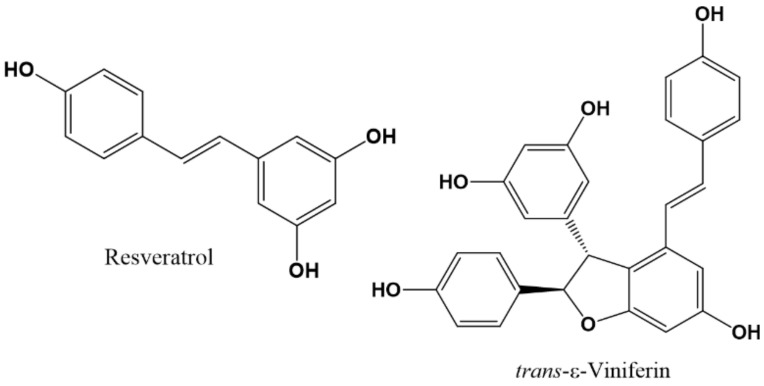
Chemical structures of active stilbenes on GABA_A_ receptor.

Two stilbenes, resveratrol and its dimer *trans*-ε-viniferin (Figure 3), have been investigated as potential modulators of GABA-Rs [142]. The effects of these two compounds were studied on different subtypes of GABA_A_R expressed in *Xenopus laevis* oocytes using the two-electrode voltage clamp technique. These compounds have different activity patterns. For example, when resveratrol is applied alone, it induces a current of 22 nA in the α_1_β_2_γ_2L_ subtype of the GABA_A_Rs (responsible for sedative and anticonvulsant effects) but has no effect on subtypes α_5_β_3_γ_2L_ (mediate learning and memory processes) and α_2_β_2_γ_2L_ (anxiolytic and muscle relaxing effects); it also produces, in a dose-dependent manner, positive modulation of the GABA-induced current (I_GABA_) in the α_1_β_2_γ_2L_ receptor, with an EC_50_ of 58.24 μM. In the case of *trans*-ε-viniferin, it negatively modulates the I_GABA_ in the three subtypes of receptors, with IC_50_ values of 5.79, 19.08 and 21.05 μM for α_1_β_2_γ_2L_, α_2_β_2_γ_2L_ and α_5_β_3_γ_2L_ subtypes, respectively. In the case of resveratrol, it effect was not sensitive to the BZD antagonist flumazenil; the effects of *trans*-ε-viniferin on α_1_β_2_γ_2L_ and α_2_β_2_γ_2L_ receptors were also not sensitive to flumazenil, but in the case of the α_5_β_3_γ_2L_ subtype, the effect was not sensitive to the inverse agonist L-655,708. The authors concluded that both compounds modulate the GABA-induced current in GABA_A_Rs by different mechanisms. In the case of *trans*-ε-viniferin, its effects are subtype selective; the authors suggested the study of different analogues of this compound for increasing the selectivity of the α_5_β_3_γ_2L_ GABA_A_R to increase the learning process [142]. Because these subunits are present mainly in the hippocampus, the inhibition of these receptors could be of interest in different pathologies such as dementia, schizophrenia, and Down syndrome [143].

In a complementary study, Li et al., (2017) analysed the effect of resveratrol on a kainic acid-induced epilepsy model in rats. In the acute phase, resveratrol reversed the silent and chronic phases of epilepsy, up-regulated the expression of the hippocampal kainate glutamate receptor, and down-regulated the GABA_A_-α_1_R. Furthermore, in the chronic phase resveratrol treatment inhibited the increased glutamate/GABA ratio in the hippocampus induced by kainic acid [144]. The hippocampus is vulnerable to epilepsy-induced injury, especially in the CA1 region [145]; resveratrol was previously demonstrated to protect against kainic acid-induced neuron death in both the CA1 and CA3a regions in a temporal lobe epilepsy model [146]. The authors concluded that the antiepileptic effects of resveratrol may be attributed in part to the reduction of glutamate-induced excitotoxicity and the enhancement of GABAergic inhibition [144].

**Figure 4:**
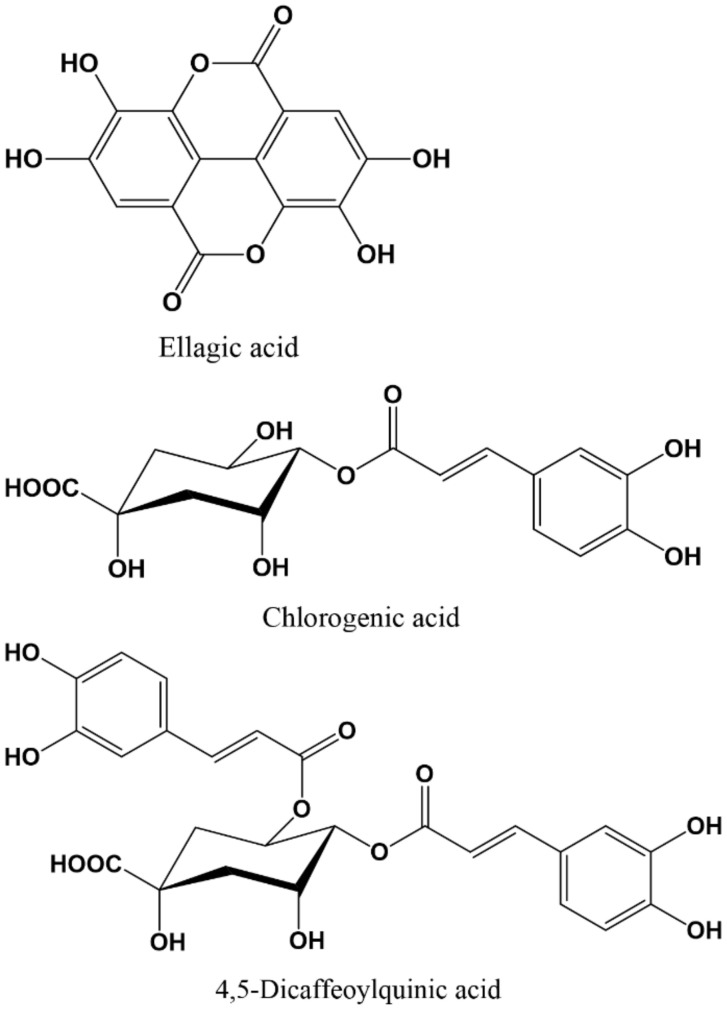
Chemical structures of active some phenol derivatives on GABA_A_ receptor.

Yaşar et al., (2013) analysed a hydroalcoholic extract of *Hypericum origanifolium* Willd (Hypericaceae) on behavioral parameters and pain perceptions of mice. The extract (50, 100, and 250 mg/kg, p.o.) induced antidepressant-like, anxiolytic-like, and antinociceptive activities after acute administrations. Moreover, the anxiolytic effect was antagonized by flumazenil, indicating the participation of the GABA_A_-BZD receptor complex. The major constituents of the extract found in the phytochemical analysis were low amounts of hyperforin, hypericin, rutin, and chlorogenic acid (Figure 4), which were considered by the authors as the possible active principles [147].

Ellagic acid (Figure 4), was established as antioxidant and anti-inflammatory, demonstrating neuroprotective effects [148]. However, to ascertain its possible anxiolytic-like effects it was studied in different animal models, corroborating the implication of the GABAergic system for the antianxiety-like effect [149]. Ellagic acid (25, 50 and 100 mg/kg, p.o.), was tested in mice (elevated plus-maze test) and, unlike diazepam, anxiolytic doses did not prolong the duration of sodium thiopental-induced loss of the righting reflex, which indicates that ellagic acid has no hypnotic effects. Its anxiolytic effect (25 mg/kg, p.o.) was antagonized by picrotoxin pre-treatment (a non-competitive GABA_A_R antagonist) and flumazenil, but not by other antagonists, demonstrating that acute and chronic administration of ellagic acid to mice has an antianxiety-like effect, with implications for the GABAergic system [149].

When Sheng et al., (2020) studied the mechanisms and active compounds in the Chinese medication (Dengzhan Shengmai) used for the treatment of cerebrovascular diseases such as chronic cerebral hypoperfusion, they observed a significant regulatory effect on glutamate and GABA-related proteins. Chemical analyses verified that 4,5-dicaffeoylquinic acid (Figure 4) and scutellarin (flavonoid), both present in this compound, could simultaneously affect the GABAergic and glutamatergic synaptic metabolism as well as the related receptors; on one hand, the α_1_ subunit of GABA_A_ was implicated. On the other hand, the 4,5-dicaffeoylquinic acid enhances the expression of the GABA_A_ receptor (α_1_β_2_γ_2_), maintains the balance of excitatory and inhibitory synaptic metabolism between glutamate and GABA, regulates glutamatergic and GABAergic synapses, and increases GABA-induced activation of GABA_A_ receptor (α_1_β_2_γ_2_). All of these results point to 4,5-dicaffeoylquinic as a GABAergic compound of high interest for future studies [100].

**Figure 5:**
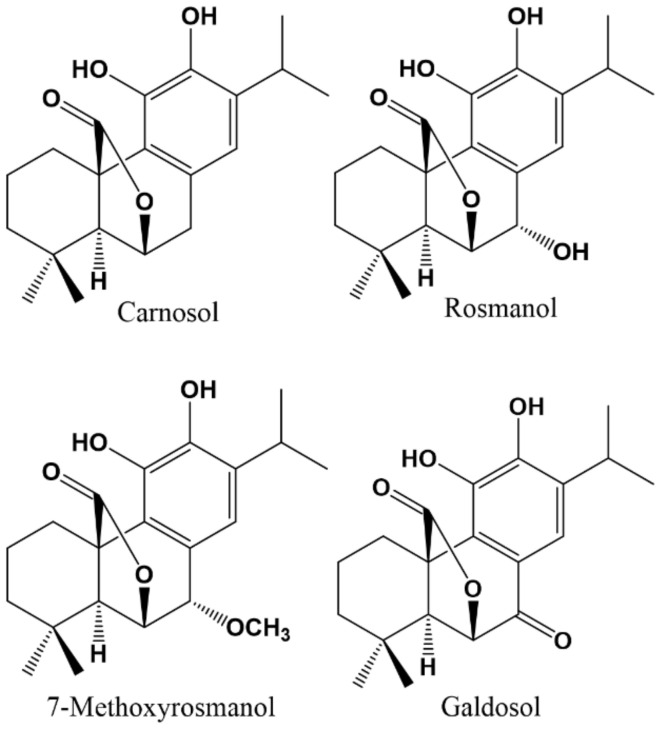
Chemical structures of active phenol-diterpenes on GABA_A_ receptor.

Abdelhalim et al., (2014) isolated two diterpene phenolics from *Salvia triloba* L. (Lamiaceae) which were identified as rosmanol and carnosol (Figure 5). They investigated their effects on human recombinant α_1_β_2_γ_2L_ receptors expressed in *Xenopus laevis* oocytes. The results obtained indicate that carnosol had no activity on these GABA-Rs when administered alone, but inhibited currents due to 100 µM GABA (IC_50_ 80.11 µM). Therefore, it is considered a non-competitive GABA blocker more effective at high doses of GABA. Rosmanol showed a biphasic mode of action, positively modulating the effect of GABA at low concentrations and inhibiting the response of GABA at high concentrations. Consequently, rosmanol has two different sites of action in the GABA receptor complex. The two phases at α_1_β_2_γ_2L_ GABA_A_Rs could be mediated by two different mechanisms: first of all, the positive modulation of the GABA response by rosmanol at α_1_β_2_γ_2L_ GABA_A_Rs was sensitive to flumazenil (only with low concentrations of GABA), indicating the involvement of a high affinity benzodiazepine binding site that requires the γ subunit. In the second phase (inhibition), the effect of rosmanol was not modified by flumazenil with or without a γ subunit, indicating this subunit is not a requirement. Therefore, the inhibitory phase is not mediated via the high-affinity flumazenil sensitive BZD site but rather occurs via the low-affinity BZD binding site [150]. The same authors [151] isolated three compounds from *Rosmarinus officinalis* L. (Lamiaceae): two flavonoids (cirsimaritin and salvigenin) and rosmanol, which were investigated in different mouse experimental models to determine the effect on modulation of GABA_A_Rs in vivo. Although positive modulation of α_1_β_2_γ_2L_ GABA_A_Rs by rosmanol was sensitive to flumazenil as previously described in vitro, when tested in vivo, the anxiolytic effect exerted by rosmanol was not antagonized by flumazenil. However, it was significantly decreased by pentylenetetrazol, which concords with a possible mechanism through GABA_A_Rs, even though not in the high affinity benzodiazepine binding site. Taken together, these results can be considered of significant interest to widen our knowledge of new active compounds on GABA_A_Rs, especially rosmanol, since some structural analogues such as 7-methoxyrosmanol and galdosol (Figure 5) have been described as ligands of the BZD-Rs, demonstrated by the inhibition of ^3^H-flumazenil binding to the BZD-R with IC_50_ values of 7.2 and 0.8 µM, respectively [152].

### 2.5. Discussion and Conclusions

**Figure 6:**
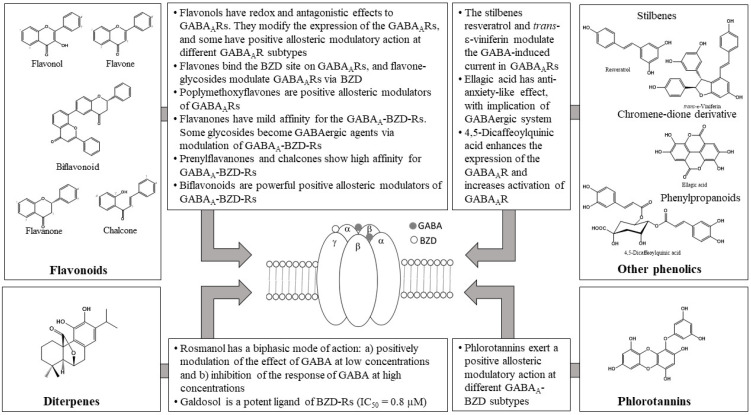
Scheme summarizing the main GABAergic effects of phenolics.

Phenolics, in special flavonoids and phlorotannins, have a proven antioxidant activity, but their pharmacological effects are not only due to this property, since they regulate different cellular functions binding directly to different targets, such as enzymes, receptors or transcription factors. Figure 6 summarizes the main effects of phenolics on the GABAergic system. 

In vivo studies revealed that flavonoids were mostly partial agonists of GABA_A_Rs, whereas only a few flavonoids seem to possess antagonistic effects. This partial agonism, at effective anxiolytic doses, was not usually accompanied by sedative or myorelaxant side effects [32]. Flavonoids may act either negatively, positively, by neutralizing GABA_A_Rs, or directly as allosteric agonists [31]. Therefore, they become potential candidates as pharmacological GABA_A_R targets for CNS pathologies involving dysfunctions of this inhibitory system. GABA_A_Rs are relevant targets of different flavonoids, such as baicalin, apigenin, chrysin, and amentoflavone [47,83,99,121]. Otherwise, quercetin has been widely studied due to its antagonistic actions on GABA_A_ρ_1_R, although it has not yet been established whether it is due to a redox mechanism, an allosteric interaction, or both. Currently, it seems that this effect is mediated through a redox-independent allosteric mechanism [70]. In the case of myricetin, it enhances GABA_A_R activity [74] and restores the expression of GABA_A_R and GAD-6, as well as the glutamate/GABA balance [129]. Regarding flavones, they also have an effect on GABA_A_Rs such as baicalin and wogonin, which have the capacity to bind to the GABA_A_-BZD receptor [90]. Luteolin activates GABA_A_Rs [79], but also shows negative modulatory effects on both recombinant and endogenous GABA_A_Rs [80], whereas apigenin activates the chloride ion channel [77] and though its glucosides modulate the GABA_A_R via BZD-binding site [104]. In regard to the dihydro-derivatives (flavanones), the methylated compounds naringenin 7-*O*-methyl ether and naringenin 7,4′-dimethyl ether naringenin) exert their activity not only on the GABA_A_-BZD receptor but also via an antagonism on the glutamatergic system, which differs from the original compound naringenin [132]. 

Chalcones, of all the structural variations of flavonoids, probably could be the ones of greatest interest given that isoliquiritigenin showed a 65 times higher affinity for GABA_A_-BZD receptors than diazepam, with a dissociation constant of 0.4 nM. The effects were blocked by flumazenil and ZK-93426, indicating an action of isoliquiritigenin by a positive allosteric modulation of GABA_A_-BZD receptors [117]. Other groups of interest are the biflavonoids, since mesuaferrone B, agathisflavone and rhusflavone showed a high potency as competitive inhibitors of flumazenil binding (Ki of 0.280, 0.091, and 0.045 μM, respectively). The most relevant of them, rhusflavone, decreases sleep latency and increases sleep duration sleep via the positive allosteric modulation of GABA_A_-BZD receptors, where the presence of a conjugated ketone and C6-C8” linkage may be essential for this effect [120]. Regarding the metoxyflavones, 5,7,2′,4′-tetrahydroxy 6,5′-dimethoxy had the higher potency (IC_50_ = 0.1 µM) in binding affinity to GABA_A_R, whereas the other compounds tested gave IC_50_ values >2.4 µM. 

The QSAR studies on the structure-activity relationship of a great number of natural and synthetic flavonoids and their BZD binding affinity showed that electronegative groups into the C-6 and C-3′ on the flavone increases their affinity with the BZD binding site and the presence of a 2′-hydroxyl on the flavonoid is also relevant for its BZD binding affinity [26,32,123]. The substitution with glycosidic forms (e.g., rutin, hesperidin and eriocitrin) or the presence of prenyl substitutions (e.g., xanthohumol, isoxanthohumol, and 8-prenylnaringenin) are of interest, because these groups of flavonoids can modify their effect on GABA modulators. For example, on one hand, rutin modifies GABAergic neurotransmission without acting on BZD-R; on the other hand, 6-prenylnaringenin may be a positive allosteric modulator at the α+ β- binding interface as well as a null modulator at the flumazenil-sensitive α+ γ_2_-binding interface [107]. All of these data taken together point out that flavonoids can be considered of interest as GABAergic agents. The effects on GABA_A_R can justify the anticonvulsive effects of quecetin and viscosine, or the anxiolytic effects of chrysin. Of particular interest is that some chalcones and biflavonoidshave different compounds relevant for their potentiality as GABA_A_-BZD receptor modulators, isoliquiritigenin and rhusflavone being examples.

Another phenolic of interest as a GABAergic agent is the diterpene galdosol, which showed the higher binding activity (IC_50_ = 0.5 µM) of the different diterpene-phenolics assayed. 

With regard to phlorotannins, the number of compounds studied is more limited than that of flavonoids, and only some of the compounds analysed showed relevant activity. In general, phlorotannins enhanced the activity of GABA_A_-BZD receptors and increased GABA_A_R-mediated inward current in cells and increased the amplitude of GABA_A_-BZD receptors in primary cultured neurons, these effects being blocked by flumazenil. These results corroborate that phlorotannins are positive allosteric activators of GABA_A_-BZD receptors, which justifies their mechanism as sedative-hypnotics, such as dieckol or triphlorethol [138,139,140]. In the case of phlorotannins, there is a relevant clinical trial in which a phlorotannin supplement increased sleep duration scores vs. placebo and decreased wakefulness after sleep as well as the total wake time. This clinical trial supports the potentiality of these compounds as sleep-promoting drugs for future studies, since there is sufficient evidence and the mechanism is clear [141]. However, there are no clinical trials on phlorotannins or flavonoids pointing out a clear GABAergic implication. Clinical data available targeted diabetes [153,154], obesity [155,156] and cognitive functioning [157]. In diabetes, the results are divergent: on the one hand, Lee and Jeon [153] found that, compared with the placebo group, the dieckol group showed a significant decrease in postprandial glucose levels after 12 weeks; on the other hand, however, Murray et al. [154] reported no lowering effects on postprandial glucose or insulin responses compared with the placebo, using neither low (500 mg) nor high (2000 mg) doses of polyphenol-rich brown seaweed (*Fucus vesiculosus*) extract. Regarding obesity, Baldrick et al. [156] established that consumption of seaweed polyphenols decreased DNA damage only to a modest extent in obese individuals, without marked effects on the clinical mediator of inflammation. Haskell-Ramsay et al. [157] examined the impact of a brown seaweed extract (polyphenol content >20%) on cognitive function and reported a significant improvement in accuracy of digit vigilance and choice reaction time tasks, although these effects could not be associated with any specific mechanism despite a modulatory effect in cognition.

Although phenolics are common in edible plants and contribute to human health through diet, these compounds are not exempt from undesirable effects and sometimes even clear toxicity. Of them, the principal group of compounds are the flavonoids, but other groups of phenolics are present in food and plants, such as coumarins, phenylpropanoids, quinones and hydrolysable tannins. These compounds usually exert many positive effects on health through their modulatory effects on different enzymes, mediators, signalling routes, and transcription factors such as protein and lipid kinase signalling cascades. Unfortunately, they may also produce negative effects due their pro-oxidant activities, such as the possible deleterious effects on cells; they can even exert deteriorating effects during development of cells and tissues. In addition, they may be responsible for estrogenic effects and be potentially carcinogenic. Also, they can interact with other drugs used for different diseases and modify their pharmacological effects; for example, tannins could be anti-nutrients or coumarins, anticoagulants [158,159,160].

In conclusion, we can establish that in the case of free aglycones, the degree of oxidation is not relevant, but the glycosylation and methylation of some hydroxyls (especially in C_6_ and/or C_8_) can modify their effects. For example, the rutinoside of quercetin has GABAergic activity without implication of BZD receptors. In the case of flavones, glycosylation modifies the GABA effect although it is maintained, e.g., baicalin vs. baicalein (aglyone). In the case of flavones, methylation could increase GABA activity, with some methoxy-derivatives (specially in C_6_ and/or C_8_) exerting higher effects than the original compounds. In chalcones where the C ring is open, activity is maintained, isoliquiritigenin having the best binding affinity on GABA_A_R. Another interesting group of flavonoids are the dimeric forms, because all tested molecules have a high binding affinity. In the case of phlorotannins, it is clear that compounds including a 1.4-dioxane group have higher binding affinity than equivalent compounds without it (eckol vs. triphlorethol-A), in which the higher molecular weight, the lower the binding affinity, e.g., eckol vs. dieckol. Phlorotannins have different molecular structures in which monomeric units are combined via different linkages into oligomers, including a relevant number of free phenolic hydroxyl radicals. These structural features could explain free radical scavenging capacities, but with the current data available, we cannot establish a relationship between the antioxidant and GABAergic activities [135,161,162].

Therefore, in conclusion, after the evidence summarized in the present review, some phenolics such as galdosol, isoliquiritigenin, rhusflavone, agathisflavone, dieckol, triphlorethol-A, among others, deserve to be considered as future subjects of research on new anticonvulsant and anxiolytic drugs (flavonoids) or sedative agents (phlorotannins).

Future research should aim at delving into these more active compounds and once their mechanism is known, future clinical trials must be developed. Due to the difficulty of carrying out these studies, the general recommendation for these trials is to focus them on extracts enriched in the compounds highlighted above, similar to those existing for brown algae, but also establishing specific criteria for pathologies of the nervous system, such as insomnia and anxiety, in which the role of the GABAergic system has been clearly established.

## Figures and Tables

**Table 1:** Flavonols with relevant GABAergic effects.

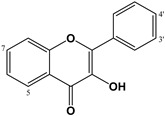 Flavonol		
Compound	Properties	Reference
Quercetin (5,7,3′,4′-tetrahydroxy)	Quercetin acts as a negative allosteric GABA_A_R modulator with antipsychotic activity. These results justify further therapeutic development of the excitatory-inhibitory imbalance disorders.	[69]
	Quercetin antagonistic actions on GABA_A_ρ₁Rs are mediated through a redox-independent allosteric mechanism.	[70]
	GABA_A_α_5_R could be a mechanism for reducing seizure severity (at anticonvulsive doses) or even be used a marker of seizure severity.	[71]
	Quercetin and its glycosides (rutin and isoquercitrin) are partially responsible for the anxiolytic and sedative-like effect of *Tilia americana* var. *mexicana* through the GABA/BZD and serotoninergic 5-HT_1A_ receptors.	[72]
Fisetin (7,3′,4′-trihydroxy)	Treatment with fisentin can delay or correct neuropathic hyperalgesia and allodynia in mice with type 1 diabetes. The analgesia caused by fisetin may be linked with its antioxidant activity. Spinal GABA_A_Rs are likely rendered as downstream targets.	[73]
Myricetin (3,5,7,3′,4′,5′-pentahydroxy)	Myricetin enhances GABA_A_R activity via the calcium channel/Ca^2+^/calmodulin-dependent protein kinase II dependent mechanism, which is distinctively different from that of most existing BZD-binding site agonists of GABA_A_R.	[74]
Viscosine (5,7,4′-trihydroxy-3,6-dimethoxy)	The anxiolytic and anticonvulsant actions of viscosine are likely mediated via its positive allosteric modulatory action at different GABA_A_R subtypes.	[75]
Glycosides		
Rutin (quercetin 3-*O*-rutinoside)	The anxiolytic-like effect involves GABAergic neurotransmission without implication of BZD receptors.	[76]
Rutin (quercetin 3-*O*-rutinoside) Isoquercitrin (quercetin-3-*O*-glucoside)	Leaves of *Tilia americana* var. *mexicana* have anxiolytic and sedative-like effects and its flavonoids, quercetin, rutin and isoquercitrin, are partially responsible due to the involvement of GABA/BZD and serotoninergic 5-HT_1A_ receptors.	[72]

**Table 2:** Flavones with relevant GABAergic effects. Aβ (β-amyloid), APP (amyloid precursor protein), EPM (elevated plus-maze), HSP70 (heat shock protein-70), MAPK (mitogen-activated protein kinase), REM (rapid eye movement), SW (slow wave).

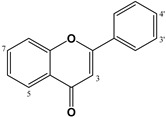 Flavone		
Compound	Properties	Reference
Apigenin (5,7,4’-trihydroxy)	Apigenin enhances pentobarbital-induced sleep behaviours through chloride ion channel activation.	[77]
	Apigenin reduces the frequency of spontaneous excitatory postsynaptic currents without affecting their amplitude, suggesting a presynaptic mechanism.	[78]
Luteolin (5,7,3’,4’-trihydroxy)	Luteolin ameliorates mechanical and cold hyperalgesia at least in part by activation of GABA_A_Rs in a flumazenil-insensitive manner and μ-opioid receptors in the spinal cord.	[79]
	Luteolin attenuates mucus overproduction and goblet cell hyperplasia in an animal asthma model at least partially by inhibition of GABA_A_R activities.	[80]
	Luteolin has negative modulatory effects on both recombinant and endogenous GABA_A_Rs and inhibits phasic rather than tonic inhibition in the hippocampus.	[81]
	The antidepressant-like effect of *Cirsium japonicum* could be mediated by luteolin through the potentiation of the GABA_A_ receptor-Cl^−^ ion channel complex.	[82]
Chrysin (5,7-dihydroxy)	Chrysin prevents anxiety-like behaviour during metestrus-diestrus in two unconditioned models. These effects were mediated by actions on GABA_A_Rs.	[83]
	Chrysin produces anxiolytic-like effects through actions on GABA_A_Rs in a model of surgical menopause in rats.	[84]
	Chrysin has more than one mechanism of action in addition to its action at the GABA_A_-BZD receptor complex, and also could be involved in its free radical scavenging abilities.	[85]
	The acute antidepressant-like effects of chrysin, similar to neurosteroids, are mediated by the GABA-binding site at GABA_A_Rs in ovariectomized rats.	[86]
Baicalein (5,6,7-trihydroxy)	The anticonvulsive effect of baicalein was mediated by the BZD binding site of GABA_A_R. The 5,7-dihydroxyl group is present in the structure of the three flavones, playing a key role in inducing convulsion-related activities.	[87]
	Baicalein promotes non-amyloidogenic processing of APP, thereby reducing Aβ production and improving cognitive performance by activation of GABA_A_Rs.	[88]
	It exhibits biphasic effects on sleep–wake regulation; decreases the SW sleep during the light period and increases SW sleep and REM sleep during the dark period.	[89]
Wogonin (5,7-dihydroxy-8-methoxy)	Wogonin is a 2.8-fold stronger ligand to the BZD binding site (K_i_ = 2.03 μM) compared to baicalein (K_i_ = 5.69 μM).	[90]
Oroxylin A (5,7-dihydroxy-6-methoxy)	In vitro studies reveal that oroxylin A blocked muscimol-induced intracellular Cl^−^ influx.	[87]
	Oroxylin A has the highest brain uptake and the highest affinity to brain tissues (In vitro tissue binding assay) compared to other flavones. This flavone, a GABA_A_ antagonist, can suppress the anxiolytic effects of other flavones present in the extract.	[91]
Glabrol (7,4’-dihydroxy-8,3’-di-isoprenyl)	Glabrol inhibits [^3^H]-flumazenil binding site to the GABA_A_-BZD receptors in the rat cerebral cortex membrane with a binding affinity (Ki) of 1.63 μM. The isoprenyl groups may play a key role in binding to GABA_A_-BZD receptors. Glabrol increases sleep duration and decreases sleep latency via a positive allosteric modulation of GABA_A_-BZD receptors.	[92]
5-Methoxyflavone	In silico studies indicate that 5-methoxyflavone exhibits good binding affinity towards GABA_A_, adenosine, glycine and NMDA receptors by H-bond interactions, justifying its hypnotic effect.	[93]
	In silico studies demonstrate a good binding affinity of 5-methoxyflavone towards GABA_A_ (α_2_ subunit-containing) and serotoninergic 5-HT_1A_ receptors by H-bond interactions.	[94]
7,8-Dihydroxyflavone	7,8-Dihydroxyflavone causes a selective reduction in the strength of GABAergic inhibition after incubation with acute cortical slices.	[95]
3-Hydroxy-2’methoxy-6-methylflavone	3-Hydroxy-2’methoxy-6-methylflavone has an anxiolytic effect without sedation or myorelaxation through positive allosteric modulation of the α_2_β_2/3_γδ_2L_ and direct activation of α_4_β_2/3_δ GABA_A_R subtypes.	[96]
2’-Methoxy-6-methylflavone	2’-Methoxy-6-methylflavone could be used as a tool to study the complex nature of the activation and modulation of GABA_A_R subtypes.	[97]
3,5,6,7,8,3’-hexamethoxy-4’,5’-methylenedioxyflavone	This methylenedioxyflavone shows anxiolytic-like activity in the EPM but locomotor responses remain unchanged.	[98]
6,7,4’,5’-dimethylenedioxy-3,5,3’-trimethoxyflavone	This methoxyflavone has anxiolytic-like activity in the EPM test involving GABA_A_R reversed by flumazenil.	[98]
3,3’,4’,5,5’,8-hexamethoxy-6,7-methylenedioxyflavone	This methylenedioxyflavone shows anxiolytic-like activity in the EPM test, with the implication of GABA_A_R, but locomotor responses remain unchanged.	[98]
6-Methoxyflavone/8-Methoxyflavone 5,7,2’,4’-Tetrahydroxy-6,5’-dimethoxyflavone	The structure-activity relationships analysis of 28 flavonoids indicate that 6-and/or 8-methoxy flavones had the most potent binding affinity to GABA_A_Rs. Of them, compound 5,7,2’,4’-tetrahydroxy-6,5’-dimethoxyflavone (IC_50_ 0.10 μM) had the higher anticonvulsant activity against chemically-induced and electrogenic seizures without myorelaxation and sedation.	[99]
**Glycosides**		
Scutellarin (scutellarein-7-*O*-glucuronide)	Scutellarin is identified by integrated metabolomics and proteomics approach as the active ingredient of Dengzhan Shengmai acting against chronic cerebral hypoperfusion due to the regulation of glutamatergic and GABAergic synapses.	[100]
Baicalin (baicalein 7-*O*-glucuronide)	Baicalin does not change intracellular Cl^−^ concentration, whereas its aglycone does. Glycosylation has a negative influence on the affinity for the BZD-binding site of the GABA_A_R.	[87]
	Baicalin activates GABAergic signalling, HSP70 and MAPKs cascades in global ischemia, which may be a mechanism underlying the baicalin’s neuroprotection.	[101]
	Baicalin inhibits SG neurons activating the BZD-sensitive GABA_A_R and/or glycine receptors, becoming a potential target for orofacial pain modulation.	[102]
Vitexin (apigenin 8-*C*-glucoside)	Vitexin has anticonvulsant effects possibly through interaction at the BZD-binding site of the GABA_A_R complex.	[103]
Isovitexin (apigenin 6-*C*-glucoside)	Isovitexin could exert its memory-enhancing and anxiolytic-like effects via GABA_A_ R modulation through its BZD-binding site.	[104]
Spinosin (apigenin 7-*O*-methyl-6-*O*-diglucoside)	Spinosin exerts anxiolytic-like effects with a mechanism of action modulated by GABA_A_ and serotoninergic 5-HT_1A_ receptors.	[105]

**Table 3:** Flavanones with relevant GABAergic effects.

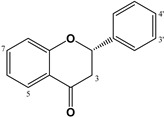 Flavanone		
Compound	Properties	Reference
Naringenin (5,7,4’-*O*-trihydroxy)	Naringenin does not produce anxiolysis by modulation of the GABA_A_Rs but it does decrease motor movements.	[106]
6-Prenylnaringenin	6-Prenylnaringenin acts as a positive allosteric modulator at α+ β− binding interface as well as a null modulator at the flumazenil-sensitive α+ γ_2_− binding interface on GABA_A_Rs.	[107]
8-Prenylnaringenin	8-Prenylnaringenin potentiated GABA-induced displacement of [^3^H] ethynylbicycloorthobenzoate radioligand binding assay in native and recombinant α_1_β_3_γ_2_, α_2_β_3_γ_2_ and α_6_β_3_δ receptors (HEK293 cells, IC_50_ of 7.3 µM).	[108]
Dihydromyricetin = ampelopsin (5,7,3’,4’,5’-*O*-tetrahydroxy)	Dihydromyricetin competitively inhibits the BZD-site [^3^H]-flunitrazepam binding site (IC_50_ 4.36 μM), suggesting the interaction with the BZD sites on GABA_A_R.	[109]
	Dihydromyricetin prevents foetal alcohol exposure consequences in pregnant rats, avoiding the alterations in physiology, behaviour, and hippocampal GABA_A_R function.	[110]
Hesperidin (hesperetin 7-*O*-rutinoside)	Hesperidin possesses anticonvulsant activity which might be mediated through the modulation of GABA-BZD receptor action.	[111]
	The antihyperalgesic effect of hesperidin combined with diosmin involves central activity partially modulated by D_2_, GABA_A_, and opioid receptors, but not serotoninergic 5-HT_1A_ receptors.	[112]
Kushenol I (3β,7,2’,4’-tetrahydroxy-5-methoxy-8-lavandulyl) (–)-Kurarinone (7,2’,4’-trihydroxy-5-methoxy-8-lavandulyl) Sophoraflavanone G (5,7,2’,4’-tetrahydroxy-8-lavandulyl)	These flavanones act as GABA_A_R modulators. They induced the I_GABA_ enhancement in *Xenopus oocytes* transiently expressing GABA_A_R with subunit composition: α_1_β_2_γ_2S._ EC_50_ are 5.0, 8.1 and 15.0 μM, respectively.	[113]
Isoxanthohumol (7,4’-dihidroxy-5-methoxy-8-isoprenyl)	Isoxanthohumol potentiates GABA-induced displacement of [^3^H]-ethynylbicycloorthobenzoate radioligand binding assay in native and recombinant α_1_β_3_γ_2_, α_2_β_3_γ_2_ and α_6_β_3_δ receptors (HEK293 cells) in a concentration-dependent manner with an IC_50_ of 11.6 µM.	[108]
Glycosides		
Eriocitrin (eriodictyol 7-*O*-rutinoside) (eriodictyol is 5,7,3’,4’-tetrahydroxy flavanone)	The antinociceptive effect of eriocitrin is blocked by naltrexone (opioid receptor antagonist) and bicuculline (GABA_A_R antagonist). Therefore, the antinociception in postoperative pain conditions could be mediated through opioid and GABA_A_Rs.	[114]

**Table 4:** Other flavonoids with relevant GABAergic effects.

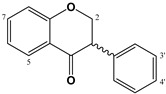	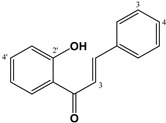	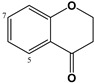
Isoflavan	Chalcone	(−)-Epicatechin
Compound	Properties	Reference
Isoflavanes		
Glabridin: (3*R*)-isoflavan-2′,4′-diol-7,8-dimethyl-6”-pyrano	Glabridin acts on GABA_A_R β subunit by a mechanism involving the M286 residue, a key amino acid at the binding site for general anaesthetics.	[115]
	Glabridin may exhibit sedative and hypnotic effects by GABA_A_R actions which potentiates GABAergic inhibition in dorsal raphe neurons. Flumazenil did not inhibit glabridin-induced potentiation.	[116]
Chalcones		
Isoliquiritigenin (4,2′,4′-trihihidroxy)	Isoliquiritigenin has higher affinity for GABA_A_-BZD receptors than diazepam. Its effect on GABA currents was blocked by flumazenil and ZK-93426). Therefore, isoliquiritigenin produces hypnotic effects by positive allosteric modulation of GABA_A_-BZD receptors.	[117]
Kuraridine (2,4,4′,6′-tetrahydroxy-2′-methoxy-5′-lavandulyl)	Kuraridine acts as a GABA_A_R modulator. It induces I_GABA_ enhancement in *Xenopus oocytes* transiently expressing GABA_A_Rs with subunit composition: α_1_β_2_γ_2S_ (EC_50_ 4.0 μM).	[113]
Xanthohumol (4,2′,4′-trihidroxy-6′-methoxy-3′-isoprenyl)	Xanthohumol potentiates GABA-induced displacement of [^3^H] the ethynylbicycloorthobenzoate radioligand binding assay in native and recombinant α_1_β_3_γ_2_, α_2_β_3_γ_2_ and α_6_β_3_δ receptors (HEK293 cells, IC_50_ of 29.7 µM)	[108]
Catechins		
(−)-Epicatechin (5,7,3′,4′-tetrahydroxy)	Epicatechin increases the basal firing rate of neurons in the globus pallidus and antagonizes the inhibitory effect of GABA. A bilateral infusion into the globus pallidus diminishes the catalepsy induced by haloperidol.	[118]
(−)-Epigallocatechin-3-*O*-gallate	Epigallocatechin gallate increases Cl^−^ influx in primary cultured cerebellar cells and decreases GABA_A_Rs α-subunit expression, whereas it has no effect on the expression of β- and γ-subunits.	[119]

**Table 5:** Biflavonoids with relevant GABAergic effects.

Biflavonoids		
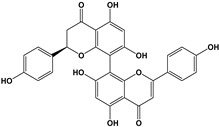 Mesuaferrone B	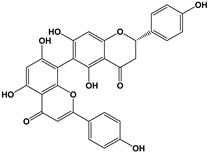 Rhusflavone	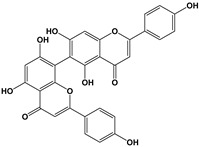 Agathisflavone
Mesuaferrone B, rhusflavone, and agathisflavone competitively inhibit the ³H-Ro 15-1788 flumazenil binding site with a K_i_ of 0.280, 0.045, and 0.091 μM, respectively. In addition, rhusflavone has sedative-hypnotic effects and is the most potent at inducing sleep. The decrease in sleep latency and increase in sleep duration seems to be due to the presence of a conjugated ketone and C6-C8” linkage in rhusflavone. It induces sleep via the positive allosteric modulation of GABA_A_-BZD receptors.		[120]

## Data Availability

Not applicable.
